# The Right to Be Counted: Unmasking First Nations and Métis Population Undercounts in Winnipeg, Manitoba

**DOI:** 10.3390/ijerph23081017

**Published:** 2026-08-04

**Authors:** Lisa Avery, Marcie Snyder, Monica Cyr, Della Herrera, Leona Star, Stephanie Sinclair, Janet Smylie, Michael Rotondi

**Affiliations:** 1 University Health Network, Toronto, ON M5G 2M9, Canada; 2 Dalla Lana School of Public Health, University of Toronto, Toronto, ON M5T 3M7, Canada; 3 Meeting Ground Consulting, Wasaga Beach, ON L9Z 1J8, Canada; 4 Aboriginal Health & Wellness Centre, 81 Higgins Ave., Winnipeg, MB R3B 3G1, Canada; 5 First Nations Health and Social Secretariat of Manitoba, 74-360 Kernaghan Avenue, Winnipeg, MB R2C 5G1, Canada; 6 Well Living House, Li Ka Shing Knowledge Institute Unity Health Toronto, Toronto, ON M5B 1X3, Canada; 7 Department of Family and Community Medicine, Faculty of Medicine, University of Toronto, Toronto, ON M5G 1V7, Canada; 8 School of Kinesiology and Health Science, York University, Toronto, ON M3J 1P3, Canada

**Keywords:** indigenous health, population enumeration, census

## Abstract

**Highlights:**

**Public health relevance—How does this work relate to a public health issue?**

- Accurate enumeration of populations is essential to population health assessment, evidence-based health services planning, and health system performance measurement.
- In Canada, enumeration of Indigenous peoples in urban areas is consistently under-estimated by two to four-fold, which obscures the health needs of a growing Indigenous population.

**Public health significance—Why is this work of significance to public health?**

- Public health can not be accurately measured, nor tracked, without knowing the size of the population in question. Baseline assessments and population-size estimates serve as a denominator for measuring service use and outcomes. Under-counting of the population size distorts health indicators and impedes comparisons across regions and over time.
- Accurate demographics of Indigenous peoples in urban areas enables the equitable distribution of resources and facilitates population specific, culturally relevant Indigenous health response.

**Public health implications—What are the key implications or messages for practitioners, policy makers and/or researchers in public health?**

- For researchers: Valid Indigenous health assessment and response will remain difficult as long as the Indigenous population size is unknown. Comparisons across regions and over time will be difficult.
- For policymakers: Amendments to the census, or a suitable alternative, such as respondent driven sampling, must be employed to allow for the accurate counting of the majority of Indigenous peoples now residing in urban areas.

**Abstract:**

**Background:** Evidence of systematic under-counting of First Nations, Inuit, and Métis (FNIM) populations living in urban centres in Canada has been documented for the previous three census rounds (2012, 2016, 2021). **Methods:** In partnership with Aboriginal Health and Wellness Centre of Winnipeg, Inc. and the First Nations Health and Social Secretariat of Manitoba, a respondent-driven sampling survey of First Nations and Métis peoples living in Winnipeg was conducted and the population living in Winnipeg was estimated using the multiplier method in conjunction with participants’ responses to census participation. **Results:** We estimated a population of 300,000 (95% CI 248,000 to 379,000) First Nations and Métis people living in Winnipeg, three times higher than the 2021 Census count of 90,535. **Interpretation:** The Canadian census continues to systematically undercount Indigenous peoples living in urban centres. Improving the accuracy and precision of Indigenous population enumeration is required for equitable health services funding and delivery. Partnerships with FNIM governing and health service organizations, addition of Indigenous identity to the short form-census participation and coverage, and improved collection methods for people living in collective dwellings or experiencing housing insecurity are recommended.

## 1. Introduction

Reliable population counts are critical for measurement of, and strategic response to, mortality, morbidity and the incidence and prevalence of disease. Accurate demographics are required for health needs assessment, health and social system performance measurement, and resource allocations, including the Canada Health Transfer. When enumeration of populations experiencing social inequities is incomplete, health disparities are commonly masked. This results in perpetuation of inequitable resource distribution and linked under-service. Durban et al. refer to this as *dysinclusion* and argue that it should be a core component of evaluating research quality—on par with bias or confounding [1].

Recent commentaries in *Science* have highlighted difficulties with population enumeration [2] and issues surrounding the provision of disaggregate data on Indigenous populations [3]. In August 2025, the United Nations Human Rights Council released a thematic report, the *Study by the Expert Mechanism on the Rights of Indigenous Peoples*, which states:

The right to data, including with regard to data collection and disaggregation, constitutes a fundamental human right for Indigenous Peoples, and data are a cultural, strategic and economic resource [4].

Research following the 2016 census showed that Indigenous peoples living in Toronto, Canada were grossly under-counted by the Canadian census [5]. Expanding on this work, our team has subsequently demonstrated that in the urban centres of London, Thunder Bay, and Kenora, Ontario, the Canadian Census under-counted the Indigenous population by a factor between two and four [6]. These findings align with patterns observed internationally, where equity-deserving populations are consistently under-counted.

In the United States, the National Advisory Committee on Racial, Ethnic, and Other Populations identified several groups as typically “harder to reach and/or enumerate include” [7], including racial and ethnic minorities, persons who mistrust the government, and people experiencing homelessness. Indigenous peoples in Canada exist at the intersections of these categories—as populations who experience ongoing colonial harms rooted in racism and whom as a result experience disproportionately higher rates of housing insecurity and homelessness [8,9] and may mistrust government institutions. As Kaneshiro [10] reported, “the political climate in which a person is embedded—particularly for persons who may feel threatened or marginalized by the government and/or the public—affects that person’s willingness to respond to the census”. Williamson et al. [11] report on the challenges of enumerating the Indigenous peoples of Australia noting significant undercount in the census data. They describe the “Indigenous data paradox”; data that focuses pejoratively on inequity yet provides insufficient context for Indigenous community planning. They argue for an updated census question based on identity with further questions eliciting information on nation and language group, as is done in the US and Aotearoa (New Zealand). Difficulties in enumerating Indigenous populations in Canada include: the Indian Act which has and continues to limit recognition of Indigenous identity; colonial policies of disenfranchisement; ongoing harms tied to self-identifying to government representatives (such as inappropriate child apprehension); disproportionate homelessness and the exclusion of Indigenous peoples from their own data governance.

The Canadian census presents specific challenges for accurately enumerating Indigenous populations. There is no question regarding Indigenous identity on the short-form census; Indigenous identity is asked only of people living in private dwellings, and only 25% of census-takers are selected to complete the long-form census. This sampling approach systematically excludes many Indigenous people, including those who are unhoused, and limits precision. Responsibility for health data management of Indigenous peoples across Canada is dispersed, limiting the utility of these administrative data to supplement the census. Indigenous Services Canada tracks data for First Nations on reserve outside of British Columbia (BC) and Inuit in Inuit Nunangat. The First Nations Health Authority (FNHA) is responsible for First Nations with status both on and off reserve in BC. Indigenous health providers and provincial and territorial health authorities may collect data on First Nations, Inuit and Métis and outside of Inuit Nunangat. A scale-up of the FNHA model across Indigenous nations would allow tracking of demographic trends within the framework of Indigenous data sovereignty [12].

The implications of under-counting extend directly to health service delivery and funding. As the Government of Canada notes, “Indigenous peoples are included in the per capita allocations of funding from the federal fiscal transfer and are entitled to access insured provincial and territorial health services as residents of a province or territory” [13]. When populations are systematically under-counted, per capita allocations fail to meet actual needs, exacerbating systemic inequities in healthcare access and quality. Moreover, these systematic undercounts cascade throughout Indigenous health data systems and surveys. The newly launched Survey Series on First Nations People, Métis and Inuit (SSFNPMI) [14] is based on the 2022 Indigenous Peoples Survey (IPS), which in turn is based on the 2021 Census questionnaire. Failure to adequately enumerate Indigenous peoples during the census leads to compounding errors in the reporting of Indigenous health data.

The objectives of this paper are to: (1) Quantify the extent of Indigenous population under-counting in the City of Winnipeg, Manitoba’s largest urban centre and home to the largest urban population of First Nations, Inuit and Métis peoples in Canada. (2) Discuss the implications of persistent undercounting of First Peoples living in Canadian cities for public health planning, resource allocation, and health equity.

## 2. Materials and Methods

Working in partnership with the Aboriginal Health and Wellness Centre of Winnipeg, Inc. (Winnipeg, MB) and the First Nations Health and Social Secretariat of Manitoba, we conducted a respondent-driven sampling population health assessment with First Nations and Métis peoples living in Winnipeg, Manitoba.

Notably, Inuk participants were invited to participate in a separate Inuit-focused study. Preliminary descriptive findings have been shared back in community reports [15,16]. Drawing on previous successful studies we used respondent driving sampling (RDS) to recruit First Nations and Métis participants 15 years of age and older who lived, worked, or received health services in the City of Winnipeg from 26 September 2023 to 7 July 2024. Sampling began with a convenience sample of 12 purposively sampled seeds, chosen to achieve diversity across social networks (age, gender, geography). Each participant was given three coupons with which to recruit. Recruitment was tracked through coupon codes (3315 coupons distributed, 1093 returned). We maintained a detailed coupon tracking log and conducted weekly data monitoring including demographics checks to identify duplicate entries. Our target sample size of 1000 participants was based on a desired precision of ±10% and design effects from previous RDS samples.

Participants were invited to complete a comprehensive health assessment survey at two sites in Winnipeg and were paid $25 to complete the survey and $10 for each successful recruitment. To estimate each participant’s probability of being included in the sample we asked the following question: *Approximately how many First Nations and Métis people do you know (i.e., by name and who know you by name) who currently live, work or use health, education or social services/programs, or access education in the City of Winnipeg?* As suggested by the STROBE-RDS guidelines, this question was used to ensure that we accurately estimated the number of connections each participant had to other members of the target population [17]. The response was used as the network degree for the calculation of RDS-II weights to estimate the proportion of the population who completed the 2021 Census [18]; calculations were performed using the RDS package in R [19,20]. The OHC survey also asked about type of residence, which allowed us to estimate the proportion of the First Nations and Métis population living in private dwellings, collective dwellings, or who were unsheltered. In keeping with our Indigenous research partnership methodology, Aboriginal Health and Wellness Centre of Winnipeg, Inc. and the First Nations Health and Social Secretariat of Manitoba co-led study design, led recruitment and survey data collection, governed all study data, and co-led the interpretation of results and publications. Project approval was provided by the Well Living House Counsel of Grandparents and community research partners. The Health Information Research Governance Committee of the First Nations Health and Social Secretariat of Manitoba reviewed and approved the project. The OHC Winnipeg study also received ethics clearance from the St. Michael’s Hospital Research Ethics Board at Unity Health Toronto (REB# 23-062). Study reporting follows the STROBE-RDS guidelines [17].

Statistics Canada conducted a Census Undercoverage Study [21] following the 2021 Census to estimate and adjust for net population undercoverage, releasing the final census estimates on 27 September 2023 which formed the basis of our comparison figures. To estimate the size of the First Nations and Métis population we used the multiplier approach [22,23]. Though attempts have been made to apply the network properties of respondent-driven sampling (RDS) to estimate total population size, these efforts have had limited success due to their lack of precision [24]. We believe the multiplier approach offers better accuracy and precision and allows for comparisons across previous studies. First, we begin with the Statistics Canada census figures and assume that the number of Indigenous *people per household*, for those living in private dwellings, can be accurately determined from the census. Second, we compute an under-reporting factor as the ratio of *expected* census completion to *reported* completion among those in private dwellings, defined as those who responded “Yes” to having completed the 2021 Census in the OHC survey. We assign confidence intervals to this ratio based on the RDS-adjusted estimate of the rate of census completion, using the RDS-II estimator. With this information, we estimate the size of the population of First Nations and Métis in private dwellings by multiplying the census figure by the under-reporting factor. Finally, we estimate the total population size (encompassing those in collective dwellings and unsheltered) by scaling up from the proportion estimated to live in private dwellings. Because the census only captures data on Indigeneity for those in private dwellings, we rely on our sample data to estimate this proportion. Full calculation details are provided in the statistical supplement (Supplemental File S1).

## 3. Results

A total of 1090 eligible adults were surveyed over 26 recruitment waves; Table 1 details the n = 13 exclusions and Table 2 describes key sample demographics. We verified that the population and sampling process were consistent with the assumptions of the Volz–Heckathorn RDS-II estimator [18]. The largest recruitment chain had 26 waves (excluding seeds) to ensure sample independence from the seeds. There was no evidence of exhaustive sampling (the average degree remained >30 in later waves), providing evidence that the population size (*N*) is sufficiently greater than the sample size (*n*) for the assumption of the RDS-II estimator requiring *N* >> *n*. The average degree was highest among the early waves, supporting the assumption that participants with the most connections have a higher probability of recruitment. Convergence plots indicated gender and age group estimates had stabilized (Supplemental Figures S1 and S2). Table 3 provides the RDS-derived estimates of the proportion of participants completing the census and living in private dwellings (21.6% and 68.9%, respectively). Based on household size and composition, we would expect 53.2% to complete the census, giving an under-reporting factor of 2.421 (95%CI 1.996, 3.058) (Supplemental Table S3). Table 4 details the population counts as reported by the 2021 Census and calculated from the OHC Winnipeg study. All calculations are shown in the statistical supplement (Supplemental File S1).

## 4. Discussion

Our best estimate of the First Nations and Métis population in the City of Winnipeg is 300,326. This is over three times the 2021 reported figure of 90,535. This aligns with previous findings based on census counts supplied in 2006, 2011 and 2016 documenting under-reporting of census counts on the order of two to four times the true population [5,6]. Despite the importance of accurate enumeration of the Indigenous population for addressing health disparities, allocating resources, and ensuring that Indigenous peoples’ right to data is upheld, systematic under-reporting persists. Under item 49 of the UN report on Indigenous rights to data Canada’s submission states that “The 2026 census of population dissemination consultation team facilitated sessions with Indigenous data users and organizations to gather their valuable insight.” (UN Human Rights Council Expert Mechanism on the Rights of Indigenous Peoples 2025). Unfortunately, this consultation process has not resulted in operational changes that would ensure broader population coverage in the 2026 census (Statistics Canada, personal communication).

The implications of this under-counting ripple throughout data collection, policy development and resource allocation. When populations are underestimated by this magnitude, per capita funding allocations fail to meet actual needs, limiting effectiveness of health systems planning, service delivery and exacerbating inequities in health care. The data problem extends beyond the census and compromises all subsequent health surveillance efforts, including the newly launched Survey Series on First Nations People, Métis and Inuit (SSFNPMI), which relies on census-based sampling frames.

To address these persistent enumeration challenges, we recommend several key changes:

- Establishment of processes for FNIM communities, governing bodies, and service organizations to co-design, access, validate, and analyze census processes and data pertaining to their local populations;
- Inclusion of Indigenous identity questions in the short-form census to capture the entire population rather than the current 25% sample;
- Implementation of enumeration strategies that include FNIM persons who are living in collective dwellings or experiencing precarious housing/houselessness.

### Limitations

Study limitations include those inherent to all RDS studies; namely that they are not probability based and instead rely on a self-reported proxy for estimating sample probability. Limitations noted by Rotondi et al. (2017) also apply to the current study: there may be recall bias regarding census completion, the population itself may have changed in the three years between the census and our study and it is possible that only a sub-sample of the Métis population has been captured in the current study [5]. To compensate for these limitations, we have tried to make conservative assumptions when estimating the population size and provided confidence intervals around our estimates which account for the variability in the RDS methodology. Even using conservative assumptions and the lower confidence limits for the population residing only in private dwellings our study shows that half the population has been missed.

## 5. Conclusions

The Canadian Census continues to vastly undercount the number of First Nations, Inuit and Métis peoples living in urban areas in Canada. Our analysis of Our Health Counts data in the city of Winnipeg shows that the size of the local First Nations and Métis population is approximately 300,000 people, more than three times larger than reported by the 2021 Census. Inaccurate enumeration contributes to inadequate allocation of health service resources and further exacerbates health inequities for local First Nations and Métis communities.

## Figures and Tables

**Table 1:** Study exclusions.

Reason for Exclusion	n
Inuit participants	3
Forgot coupon	1
Unable to reliably respond to survey questions *	5
Not Indigenous	4
Total excluded	13

* These individuals were deemed unable to reliably respond to the survey questions at the time of their interview due to unforeseen physical, cognitive, and/or mental health issues that were not identified at the time of recruitment.

**Table 2:** Key sociodemographics of OHC Winnipeg First Nations and Métis sample (n = 1090).

	Unadjusted N (%)	RDS-II Adjusted% (95% CI)	Design Effect
Identity			
First Nations	885 (81.2)	80.5 (76.5, 84.5)	2.9
First Nations/Métis	23 (2.1)	3.1 (1.4, 4.8)	2.6
Métis	182 (16.7)	16.4 (12.6, 20.2)	2.9
Gender			
Female	526 (48.3)	46.3 (41.4, 51.1)	2.7
Male	555 (50.9)	53.1 (48.3, 57.9)	2.7
Trans/Gender Diverse	9 (0.8)	0.7 (0.0, 1.6)	3.8
Age Group			
15–29	154 (14.1)	19 (15.0, 22.9)	2.9
30–44	382 (35)	38 (33.4, 42.5)	2.5
45–59	434 (39.8)	35.5 (30.9, 40.1)	2.6
60+	120 (11)	7.6 (4.8, 10.3)	3.1
Housing			
Experiencing Homelessness/Houselessness	87 (8)	7.2 (5.2, 9.1)	1.6
Multi-generational household	235 (21.6)	20.6 (16.6, 24.7)	2.8
Multi-generational/family household	155 (14.2)	13.1 (9.6, 16.6)	3.1
Single generation household	99 (9.1)	12.3 (9.7, 14.9)	1.8
Single or living with unrelated household members	510 (46.8)	46.6 (41.8, 51.4)	2.6
Don’t Know/Declined	4 (0.4)	0.2 (0.0, 0.7)	2.8
Education			
Less than high school	570 (52.3)	56.8 (52.1, 61.6)	2.6
Completed high school	275 (25.2)	29.1 (24.6, 33.6)	2.8
More than high school	244 (22.4)	14 (10.8, 17.3)	2.5
Don’t know/Declined	1 (0.1)	0 (0.0, 0.2)	3.1
LICO			
Above	36 (3.3)	3.1 (1.4, 4.7)	2.5
At or below	1016 (93.2)	94.2 (91.8, 96.7)	3.2
Don’t Know/Declined/Missing	38 (3.5)	2.7 (0.8, 4.7)	4.1
Reported network degree Median (IQR)	60 (20–200)		

**Table 3:** RDS-adjusted estimates of the proportion of the OHC Winnipeg First Nations and Métis who completed the census and lived in an Indigenous dwelling/household.

	N	RDS-II (%)	Unadjusted (%)
Completed Census			
No	520	73.7 (68.9, 78.5)	66.4
Yes	201	21.6 (17.1, 26.2)	25.7
Don’t Know	62	4.6 (2.5, 6.8)	7.9
Dwelling Type			
Collective/Unsheltered	306	30.7 (26.7,34.8)	28.1
Private	783	68.9 (64.8, 73.0)	71.8
Don’t Know/Declined	1	0.4 (0.0, 1.4)	0.1

**Table 4:** Comparison of 2021 Census figures with Our Health Counts (OHC) population estimates for the Non-Inuit Indigenous population of Winnipeg, rounded to the nearest thousand.

First Nations and Métis Population in Winnipeg	2021 Census Figure	OHC Winnipeg Private Dwelling Population Estimate (95% CI)	OHC Winnipeg Total Population Estimate
Total First Nations and Métis Population	90,535	219,000 (181,000 to 277,000)	300,000 (248,000 to 379,000)
First Nations and Métis Children	23,955	58,000 (48,000 to 73,000)	79,000 (66,000 to 100,000)
First Nations and Métis Adults	43,495	161,000 (133,000 to 204,000)	221,000 (182,000 to 279,000)

## Data Availability

The datasets used for analyses during the current study are not publicly available due to data sharing agreements between the community research partners and investigators.

## Supplementary Materials

The following supporting information can be downloaded at: https://www.mdpi.com/article/10.3390/ijerph23081017/s1, Figure S1: Convergence plots for the key demographic indicators of gender and age group, Figure S2: Weekly recruitment and degree reports. The number of participants recruited each week is shown with the grey bars indexed to the left-hand y-axis. The mean and median reported degree of the new recruits for each week is shown by the red and grey lines, with a log-based scale on the right-hand axis, File S1: Statistical Supplement.
