# Supplementary material for: The Right to Be Counted: Unmasking First Nations and Métis Population Undercounts in Winnipeg, Manitoba
Source: Int J Environ Res Public Health. 2026 Aug 4;23(8):1017. doi: 10.3390/ijerph23081017 (PMC13512762; doi:10.3390/ijerph23081017)
Supplement: Supplementary file 1 [file ijerph-23-01017-s001.zip › ijerph-4383108-supplementary.pdf]

RDS Supplement

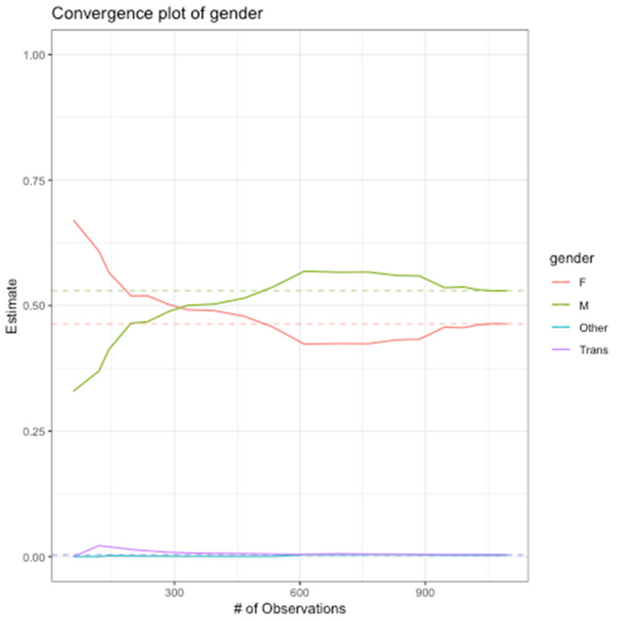

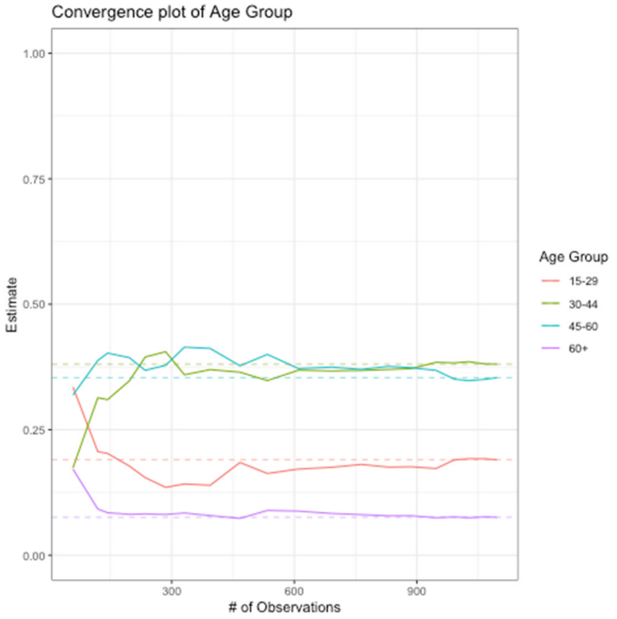

Figure S1: Convergence plots for the key demographic indicators of gender and age group

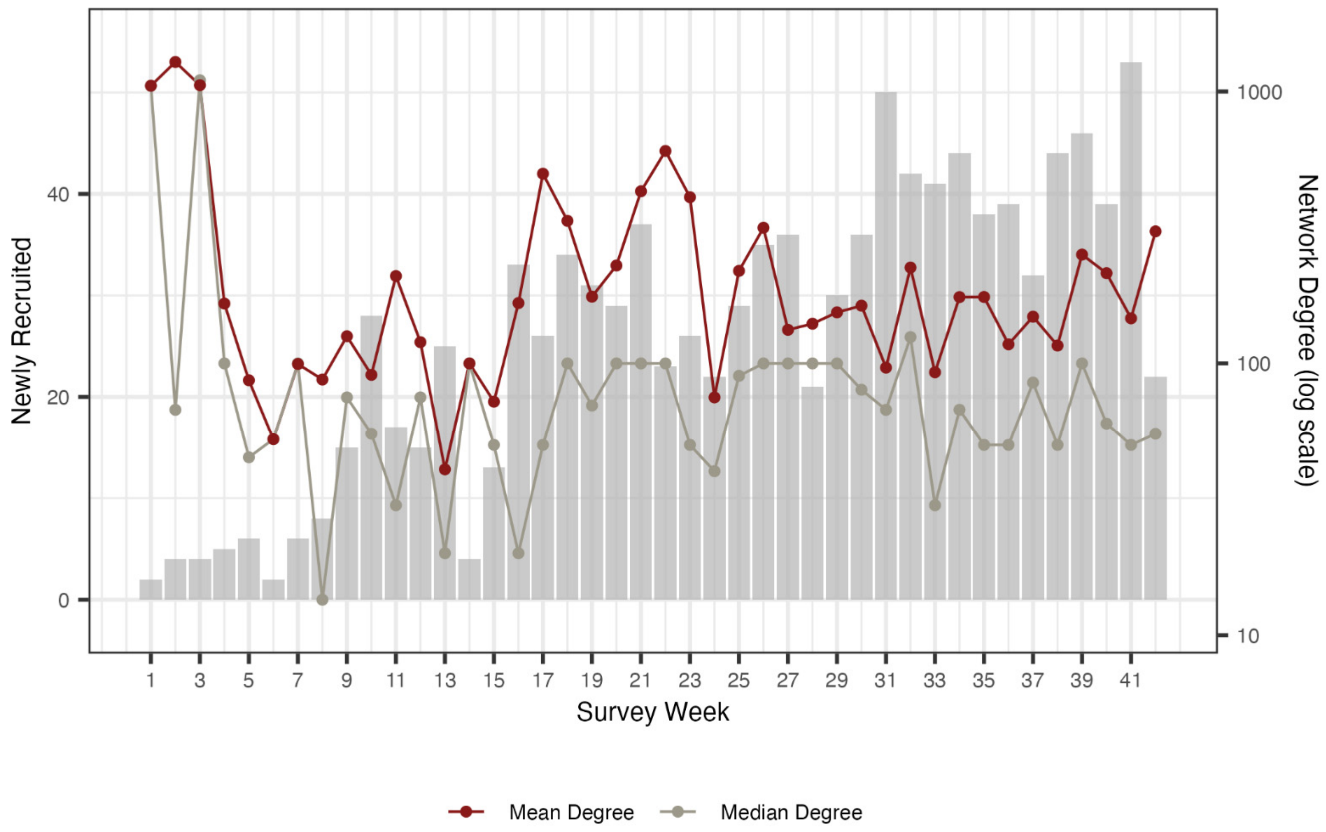

Figure 2: Weekly recruitment and degree reports. The number of participants recruited each week is shown with the grey bars indexed to the left-hand y-axis. The mean and median reported degree of the new recruits for each week is shown by the red and grey lines, with a log-based scale on the right-hand axis.

File S1. Statistical Supplement

Table S1: Indigenous population of Winnipeg, City [Census subdivision], Manitoba, as reported by Statistics Canada for the 2021 Census of Population (Statistics Canada 2023), excluding 455 participants responding as Inuk.

First Nations and Métis Indigenous Population of Winnipeg

	Census Figure
Adults and Children	90,535
Adults	66,585
Children	23,955
Private Indigenous Dwellings	43,495
Average Indigenous Household Size**	2.6*

Notes:
* Private dwelling refers to a separate set of living quarters with a private entrance either from outside the building or from a common hall, lobby, vestibule or stairway inside the building. The entrance to the dwelling must be one that can be used without passing through the living quarters of some other person or group of persons. *Source: Standards, data sources and methods, Statistical Units*

** *The Average Household Size includes both Indigenous and non-Indigenous adults and children in Indigenous households.* An Indigenous household is one of the following: i) a non-family household in which at least 50 per cent of household members self-identified as Indigenous people; or ii) a family household that meets at least one of two criteria: a) at least one spouse, common-law partner, or lone parent self-identified as an Indigenous person; or b) at least 50 per cent of household members self-identified as Indigenous people. An Indigenous person is anybody identifying as an Indigenous person (question 24 on the 2021 Census long-form questionnaire), or who is a Treaty Indian or Registered Indian (question 26), a member of an Indian Band/First Nation (question 27), a registered member of a Métis organization or settlement (question 28) or enrolled under, or a beneficiary of, an Inuit land claims agreement (question 29). *Source: Footnote 2, Table of Dwellings/Households*

Population Calculation Methodology

First, we calculate the population estimate for the people *living in private dwellings* as follows:

Estimated population in private dwellings = Census reported figure x under reporting factor

Where the Under-reporting factor (URF) is the ratio of the proportion of the population *expected* to complete the census to the proportion *who reported* completing the census, for those living in private dwellings.

Expected Census Completion

This applies the census estimate of the household size and then estimates the number of adults per Indigenous household.

Proportion of adults in households: $\frac{66{,}585}{90{,}535} = 0.735$

Total number of adults (including Indigenous + non-Indigenous) per household is $2.6 \times 0.735 = 1.911$

The expected probability of completing the census for any adult in an Indigenous household is:

$$p_{expected} = \frac{1}{1.911} = 0.523$$

Observed Census Completion

The RDS estimates of census completion for the n=783 participants who reported living in private dwellings are shown in Table S2. Between 17-26% of the sample reported completing the 2021 census; we use this confidence interval to determine a range for our under-reporting factor and then our estimate of the population in private dwellings.

Table S2: RDS-adjusted estimates of the proportion of the OHC Winnipeg First Nations who completed the census and lived in an Indigenous dwelling/household (included in Table 3 in the text)

Completed Census	N	RDS-II (%)	Unadjusted (%)
Don't Know	62	4.6 (2.5, 6.8)	7.9
No	520	73.7 (68.9, 78.5)	66.4
Yes	201	21.6 (17.1, 26.2)	25.7

As completion of the Census is required by law, we suspect that some respondents who selected Don't Know may have selected this category out of fear of reporting that they had not completed the required questionnaire. For this reason, we focus our analysis on those who reported completing the Census; recognizing that many participants who selected the Don't Know category may not have completed the Census.

Under-Reporting Factor

This is the ratio of the proportion we expect to complete the census to the proportion who reported completing the census.

Table S3: Estimate of the under-reporting factor, with 95% confidence intervals from the RDS-adjusted estimate of the rate of census completion

Estimate	Calculation	Under reporting factor
Point Estimate	0.523 / 0.216	2.421
Lower Bound	0.523 / 0.262	1.996
Upper Bound	0.523 / 0.171	3.058

Using the confidence intervals around the under-reporting factor, the number of Indigenous people, adults and children per household from the 2021 census, and the number of Indigenous households of 43,495, we estimate the population size *in private dwellings*, with 95% confidence.

Population in Private Dwellings:

Proportion in Private Dwellings

Table S4: Percentage of respondents from OHC Winnipeg living in private and collective dwellings (included in Table 3 in the text).

Dwelling Type	N	RDS-II (95% CI)	Unadjusted (%)
Collective/Unsheltered	306	30.7 (26.7,34.8)	28.1
Don't Know/Declined	1	0.4 (0.0, 1.4)	0.1
Private	783	68.9 (64.8, 73.0)	71.8

The total population size is estimated by dividing the number in private dwellings by the *upper bound* of the RDS-adjusted proportion of people living in private dwellings. The *upper bound* (0.730) of the RDS-adjusted confidence interval is used to ensure a conservative model for the total population size.

Total Population Estimate:

Table S5: Total estimate of the First Nations and Métis population of Winnipeg in 2021, rounded to the nearest thousand.

First Nations and Métis Population of Winnipeg	OHC Private Dwelling Population Estimate (95% CI)	OHC Total Population Estimate (95% CI)
**Adults & Children**	219,000 (181,000–277,000)	300,000 (248,000–379,000)
**Children**	58,000 (48,000–73,000)	79,000 (66,000–100,000)
**Adults**	161,000 (133,000–204,000)	221,000 (182,000–279,000)
